# Advanced Neuroimaging and Emerging Systemic Therapies in Glioblastoma: Current Evidence and Future Directions

**DOI:** 10.3390/biomedicines13112597

**Published:** 2025-10-23

**Authors:** Ilona Bar-Letkiewicz, Anna Pieczyńska, Małgorzata Dudzic, Michał Szkudlarek, Krystyna Adamska, Katarzyna Hojan

**Affiliations:** 1 Greater Poland Cancer Centre, 15 Garbary St., 61-866 Poznan, Poland; ilonabar@interia.pl (I.B.-L.); apieczynska@ump.edu.pl (A.P.);; 2 Joseph Strus Municipal Hospital in Poznan, Department of Oncology, 3 Szwajcarska St., 61-285 Poznan, Poland; 3 Poznan University of Medical Sciences, Department of Occupational Therapy, 6 Swięcickiego St., 60-781 Poznan, Poland; 4 Joseph Strus Municipal Hospital in Poznan, Department of Neurology, 3 Szwajcarska St., 61-285 Poznan, Poland; 5 Department of Ecology and Anthropology, Institute of Biology, University of Szczecin, 13 Wąska St., 71-415 Szczecin, Poland; michal.szkudlarek@usz.edu.pl; 6 Poznan University of Medical Sciences, Department of Electroradiology, 15 Garbary St., 61-866 Poznan, Poland

**Keywords:** glioblastoma, multiparametric MRI, PET tracers, radiomics, artificial intelligence, temozolomide, CAR-T cells, MGMT promoter methylation, targeted therapy

## Abstract

Despite technological progress, glioblastoma (GBM) continues to confer dismal prognoses. Modern neuroimaging methods are assuming an ever greater role in diagnosing and monitoring brain tumors. This review shows current neuroimaging approaches and systemic therapeutic strategies for glioblastoma, with a focus on emerging and innovative treatments. Advances in multiparametric magnetic resonance imaging—MRI (diffusion, perfusion, and spectroscopy) and novel positron emission tomography (PET) tracers, complemented by radiomics and artificial intelligence (AI), now refine tumor delineation, differentiate progression from treatment effects, and may help predict treatment responses. Maximal safe resection followed by chemoradiotherapy with temozolomide remains the standard, with the greatest benefit seen in O^6^-methylguanine DNA methyltransferase (MGMT) promoter-methylated tumors. Bevacizumab and other targeted modalities offer mainly progression-free, not overall survival, gains. Immune checkpoint inhibitors (e.g., nivolumab) have not improved survival in unselected GBM, while early multi-antigen CAR-T (chimeric antigen receptor T-cell) strategies show preliminary bioactivity without established durability. While actionable alterations (NTRK fusions and BRAF V600E) justify selective targeted therapy trials, their definitive benefit in classical GBM is unproven. Future priorities include harmonized imaging molecular integration, AI-driven prognostic modeling, novel PET tracers, and strategies to breach or transiently open the blood–brain barrier to enhance drug delivery. Convergence of these domains may convert diagnostic precision into improved patient outcomes.

## 1. Introduction

Brain tumors pose a substantial clinical challenge because of their aggressive behavior, therapeutic resistance, and the profound impact they exert on patients’ neurological functions.

Gliomas are among the most common primary malignant tumors of the central nervous system (CNS) and represent the most aggressive group of brain neoplasms. Glioblastoma (GBM)—historically termed glioblastoma multiforme—is characterized by high mortality (median overall survival is below 2 years) and limited therapeutic options. The risk of developing GBM increases with age and shows a slight male predominance [1].

Neuroimaging of the CNS plays a pivotal role in the diagnosis and management of patients with brain tumors. Advances in imaging over recent years have enabled the implementation of sophisticated technologies in brain tumor care. For the diagnosis of primary intracranial brain tumors, magnetic resonance imaging (MRI) performed before and after intravenous administration of a paramagnetic contrast agent is the modality of choice.

The development of imaging techniques—particularly over the past three decades—has markedly broadened diagnostic capabilities. Neuroimaging encompasses a set of noninvasive methods that depict both the morphology and function of the brain. In neuro-oncology these techniques are employed for therapy planning, monitoring treatment efficacy, and assessing late adverse effects. The application of advanced MRI and positron emission tomography (PET) now permits highly precise mapping of structural and functional alterations in brain tissue. Further progress is anticipated through the integration of MRI (and other imaging modalities) with artificial intelligence models: combining imaging and clinical datasets within AI frameworks may ultimately improve outcomes in patients with brain tumors.

In this paper we aim to review current neuroimaging approaches and systemic therapeutic strategies for glioblastoma, with a focus on emerging and innovative treatments.

The present narrative review has been performed to synthesize contemporary advanced neuroimaging in GBM, including diffusion/perfusion MRI, MR spectroscopy, PET, radiomics, and AI-based imaging, and emerging systemic therapies, such as concomitant chemotherapy, anti-angiogenic therapy, immune checkpoint inhibition, chimeric antigen receptor T-cell (CAR-T) therapies, targeted therapies, and multi-modal combinations. Additionally, the narrative incorporates promising technologies (for instance, MR-guided focused ultrasound for blood–brain barrier opening) insofar as they interface with systemic treatment delivery or imaging endpoints.

## 2. Materials and Methods

Given the heterogeneous and rapidly evolving nature of this field, a narrative approach was adopted to facilitate the integration of mechanistic, early-phase, and translational data with clinical studies.

We focused primarily on studies published between 2019 and 2024, reflecting the most recent progress in neuroimaging and therapeutic strategies for glioblastoma. However, earlier landmark publications with a lasting impact on clinical practice—such as the original Stupp protocol—were also included to provide essential historical and methodological context. The review was performed based on studies available in PubMed (U.S. National Library of Medicine, Bethesda, MD, USA), Scopus (Elsevier B.V., Amsterdam, Netherlands), Google Scholar (Google LLC, Mountain View, CA, USA), and ResearchGate (ResearchGate GmbH, Berlin, Germany) from January 2019 through December 2024. Additionally, reference lists of the included studies and recent reviews were searched for additional sources.

The following inclusion criteria were implemented:

(1) Original studies published in English;
(2) Adult patients with newly diagnosed or recurrent GBM;
(3) Diagnostic neuroimaging or systemic treatments were interventions of interest;
(4) Type of study: Phase I–III trials, prospective/retrospective cohorts, large cases, systematic reviews, and consensus statements or guidelines.

The exclusion criteria were as follows:

(1) Case reports on animals or in vitro studies, editorials or letters lacking primary data, conference abstracts without the complete text, and pediatric-only cohorts.
(2) A lack of neuroimaging or pharmacological intervention.

Due to the heterogeneity observed among the study designs, endpoints, and reporting methods, a quantitative meta-analysis was not performed.

## 3. Results

### 3.1. Neuroimaging Methods in Diagnosis and Monitoring

#### 3.1.1. Computed Tomography (CT)

Computed tomography (CT) is an imaging technique that generates cross-sectional images of organs, tissues, and anatomical structures. The X-ray tube—serving as the energy source—rotates around the patient, emitting a beam of ionizing radiation, while detectors collect projection data. Reconstruction algorithms then produce tomographic slices from the large volume of acquired raw data. In the evaluation of central nervous system (CNS) tumors, iodinated intravenous contrast media are commonly applied to increase conspicuity of vascular structures and well-perfused tissues relative to less vascularized regions. However, their use may be limited in patients with renal insufficiency, a history of hypersensitivity reactions to iodinated contrast media, or hyperthyroidism. Radiological appearance of GBM is often used to scan for focal changes in the brain in CT, preferably followed by contrast-enhanced CT or MRI [2].

Computed tomography (CT) is usually the first-line imaging modality in acute neurological conditions (e.g., a headache with focal symptoms and altered consciousness), as it allows for rapid detection of intracranial hemorrhage, edema, mass effects, and hydrocephalus. It can be used for initial patient stratification and in situations where MRI is contraindicated (implanted metallic devices, severe claustrophobia, and unstable clinical status). CT is superior to MRI in visualizing calcifications and acute hemorrhage, which can aid in differential diagnosis (e.g., primary CNS lymphoma vs. GBM or metastases). CT is also valuable in the perioperative setting—immediately after surgery it can detect hemorrhagic complications or severe edema before a follow-up MRI is performed.

The main limitations of CT include its lower soft tissue resolution. CT poorly differentiates GBM components (enhancing core, necrosis, and edema/infiltration), making it unsuitable for precise tumor mapping, resection planning, radiotherapy targeting, or treatment response assessments. In these domains, MRI remains the method of choice. Tumor margins and white matter infiltration are underestimated on CT, reducing the reliability of resectability assessments and radiotherapy planning. Current European Association of Neuro-Oncology and National Comprehensive Cancer Network (EANO/NCCN) guidelines base decision-making primarily on MRI and histopathological/molecular diagnosis [3].

Another limitation of CT is its low sensitivity in distinguishing progression from pseudoprogression or post-radiation changes. Ionizing radiation exposure and iodinated contrast administration are important considerations, particularly for patients requiring repeated follow-up imaging. The risks of contrast-induced nephropathy and allergic reactions further limit repeated examinations.

#### 3.1.2. Magnetic Resonance Imaging (MRI)

Magnetic resonance imaging (MRI) is a noninvasive technique performed both before and after intravenous administration of a paramagnetic contrast agent. It is the imaging modality of choice for evaluating tumors of the brain, spinal cord, and spinal canal, and it provides the best method for differentiating primary from secondary intra- and extramedullary lesions. MRI uses a strong static magnetic field and radiofrequency pulses combined with advanced computational reconstruction to generate high-resolution images of the structures under evaluation.

MRI examinations can be supplemented with proton MR spectroscopy to refine characterization of the tumor type [4]. A multiparametric MRI protocol should include pre- and post-contrast T1-weighted images, T2-weighted images, fluid-attenuated inversion recovery (FLAIR), diffusion-weighted imaging (DWI), and perfusion-weighted imaging (PWI). FLAIR is a modified T2-weighted inversion recovery sequence that suppresses the high signal of the cerebrospinal fluid (CSF), thereby improving the conspicuity of pathological changes that are hyperintense on T2 but are not free fluid—such as demyelinating foci, gliotic scars, or vasogenic edema.

During MRI, an intravenous gadolinium-based contrast agent (GBCA) is commonly administered. Elemental gadolinium is toxic; however, in clinical contrast media, it is bound in chelated complexes that are considered safe for the body. In most patients GBCAs are well tolerated, and adverse events—typically hypersensitivity reactions—are very rare. However, in patients with severe renal impairment, there is a risk of developing nephrogenic systemic fibrosis (NSF) [5].

Contrast-enhanced MRI remains the gold standard for suspected brain tumors, for initial characterization (location, extent, and enhancing component/necrosis/edema), and for post-treatment monitoring. Established sequence protocols (T1/T1c, T2, FLAIR, and DWI) allow mapping of mass effects and inform surgical and radiotherapy planning. High soft tissue resolution enables a detailed assessment of changes within contrast-enhanced and T2/FLAIR areas, as well as the detection of treatment-related complications.

MRI is commonly seen as an excellent tool for treatment planning. Integration of MRI data (including perfusion/spectroscopy and tractography) with radiotherapy planning and neuronavigation systems facilitates safer resections and more precise PTV/CTV delineation. An increasing number of studies indicate that combining multiparametric MRI with amino acid PET improves the selection of the most malignant foci for biopsy or radiation boost [6].

Limitations of MRI include difficulties in distinguishing pseudoprogression and pseudoresponses. After chemoradiotherapy and during anti-angiogenic therapy, enhancement patterns can confound interpretation. The Response Assessment in Neuro-Oncology (RANO) 2.0 criteria recommend repeated MRI scans to confirm progression during the high-risk window. Morphology and contrast enhancement alone do not always reflect the true biology of the lesion [7].

Another limitation involves heterogeneity in protocols, scanners, and acquisition parameters (particularly in PWI/MRS), which restrict comparability and generalizability of results. Therefore, standardized protocols and detailed reporting of acquisition parameters are needed [8].

In some patients (e.g., low-grade/IDH-mutant gliomas, non-enhancing lesions, or post-treatment regions), MRI alone may be insufficient to confidently differentiate progression from treatment-related changes. Evidence indicates that amino acid PET can be more accurate in distinguishing progression from radiation-induced changes, and in this specific clinical scenario, it may even outperform contrast-enhanced MRI [9]. Finally, in clinical practice MRI may be limited by patient motion, artifacts, long examination times, and restricted availability of advanced sequences.

#### 3.1.3. Diffusion-Weighted MRI (DWI)

Diffusion-weighted imaging (DWI) utilizes the microscopic Brownian motion of water molecules, which is altered by pathological processes in tissue. In gliomas, proliferating tumor cells decrease the extracellular space, limiting water proton displacement, which leads to restricted diffusion. This phenomenon is depicted on DWI as high signal intensity with corresponding low values on the apparent diffusion coefficient (ADC) map. Reduced diffusion within a lesion can therefore suggest higher tumor cellularity and, in some contexts, more aggressive biological behavior [10,11].

#### 3.1.4. Perfusion-Weighted MRI (PWI)

Perfusion-weighted MRI (PWI) is a functional technique that evaluates the capillary level cerebral blood flow, yielding valuable insights into the status of the cerebral microcirculation. Increased perfusion is typically observed in highly vascular, high-grade neoplastic lesions. PWI is used in the assessment of primary brain tumors to characterize vascularity, differentiate low- from high-grade tumors, and help delineate tumor margins relative to surrounding vasogenic edema. It also contributes to longitudinal monitoring of disease progress and treatment responses [10,11], and it plays a pivotal role in distinguishing radiation necrosis from tumor recurrence.

Glioblastoma typically appears on MRI as a heterogeneous, poorly demarcated mass on both T1- and T2-weighted images, often containing blood metabolites and necrosis and surrounded by extensive vasogenic edema. Following contrast administration, enhancement is generally vivid and heterogeneous, frequently appearing as a ring with a thick, irregular margin.

#### 3.1.5. MR Spectroscopy (MRS)

Magnetic resonance spectroscopy (MRS) is a noninvasive technique that provides in vivo information on the biochemical composition of selected tissues. It evaluates the presence and relative concentrations of metabolites within a lesion, reflecting specific elements of the tumor cellular architecture and underlying biochemical processes. By detecting signals emitted by atomic nuclei, MRS enables estimation of metabolite concentrations in both normal and pathologically altered brain tissues. The most widely used clinical implementation is proton MRS (H1MRS), which permits analysis of brain metabolites such as N-acetylaspartate (NAA), creatine (Cr), and choline (Cho). With H1MRS it is currently possible to distinguish glial from mesenchymal tumors, assist in grading, and differentiate neoplasm from encephalomalacia or vascular scar tissue.

Accurate interpretation requires familiarity with spectral appearances and individual metabolite biomarkers. Creatine tends to have relatively stable concentrations and is therefore used as an internal reference to calculate ratios (Cho/Cr and NAA/Cr) for semi-quantitative assessments. N-acetylaspartate (NAA), which is localized almost exclusively in neurons, serves as a marker of neuronal density. Decreased NAA indicates neuronal loss or displacement; thus any mass that replaces or compresses the parenchyma leads to a reduction in NAA. In contrast, NAA is typically absent in the case of brain metastases or meningiomas, making it useful for differential diagnosis. Choline (Cho) indicates myelin breakdown, and its level—especially when expressed as the Cho/Cr ratio—is associated with the tumor grade. Additional useful metabolites include inositol (a glial/astrocytic marker) and glia-specific glycine, which can aid in differentiating gliomas from metastases [12,13].

#### 3.1.6. Positron Emission Tomography–Computed Tomography (PET/CT)

Positron emission tomography combined with computed tomography (PET/CT) is an advanced functional imaging technique that assesses tissue metabolism using positron-emitting radiopharmaceuticals. In brain tumors it is employed to help define tumor extent, evaluate (or support estimation of) its grade, and guide biopsy and surgical/radiotherapy planning.

The most commonly used radioactive tracer is ^18^F-fluorodeoxyglucose (^18^F-FDG), a glucose analog labeled with fluorine. After intravenous administration, increased tracer uptake can be observed in brain tumors due to elevated glucose metabolism. A major limitation of this approach in neuro-oncology, however, is the high physiological uptake of glucose by healthy brain tissue, which reduces lesion conspicuity, impairing sensitivity and specificity for both detection and grading [14,15]. Assessing tumor recurrence after prior oncological treatment (neurosurgical/radiotherapy) is a frequent diagnostic challenge.

To better distinguish true recurrence from post-radiation effects, clinicians employ amino acid tracers that exhibit low background uptake in normal tissue, enhancing lesion conspicuity. Examples include ^11^C-methionine, ^18^F-fluoro-ethyl-L-tyrosine, or somatostatin analogs [16]. For delineating tumor margins, amino acid PET often provides higher accuracy than conventional MRI, whereas MRI retains superiority for comprehensive treatment response evaluation. PET can supplement brain MRI in the assessment of pseudoprogression after anti-angiogenic therapy: reduced radiotracer uptake—sometimes with minimal changes in lesion size—may support a treatment effect rather than true progression. An emerging development in CNS tumor imaging is hybrid PET/MRI, which integrates molecular/metabolic data with the superior soft tissue contrast, higher spatial resolution, and lack of harmful ionizing radiation inherent in CT.

#### 3.1.7. Imaging Software

Radiotherapy of the brain is a commonly used modality in the treatment of brain tumors. However, despite increasingly precise radiotherapy techniques, damage to healthy brain tissue may still occur. As a result, in recent years, growing attention has been paid to monitoring structural brain changes following oncologic treatment. To this end, modern imaging technologies are combined with advanced data analysis, sometimes in a form of radiomics and radiogenomics [17].

One of the most widely used tools is FreeSurfer, a software for magnetic resonance imaging (MRI) analysis [18]. It enables automated segmentation of brain structures, measurements of cortical thickness and the volumes of gray and white matter, and longitudinal assessments of change. Advantages of FreeSurfer (v7.1.1) include automated processing of large datasets and the ability to monitor morphological and volumetric changes over time, which is crucial for research into neuroplasticity and the long-term impact of therapy on the brain. The software can be applied to analyze different radiotherapy protocols, such as WBRT and SRS. However, accurate analysis requires high-quality MRI data, and processing may be particularly difficult in patients with extensive structural abnormalities. In radiation-altered brains, FreeSurfer’s performance can be further challenged by post-treatment distortions such as lesions or resection cavities, which may lead to segmentation errors [19] and therefore require careful quality control. Manual visual inspection, with or without subsequent manual correction, remains the gold standard for FreeSurfer quality control, although automated and semi-automated approaches (e.g., MRIQC, Euler numbers, and Qoala-T) are increasingly applied to improve reproducibility and scalability [20]. Moreover, inter-scanner variability can significantly affect FreeSurfer-derived volumetric measures—even between scanners of the same model—potentially obscuring true longitudinal brain changes unless harmonization procedures (e.g., affine voxel size correction, intensity normalization, and intracranial volume scaling) are applied [21].

Studies employing FreeSurfer have shown that patients undergoing radiotherapy experience a significant reduction in hippocampal volume. In particular, one study using FreeSurfer v7.1.1 reported a median reduction of 5 mm^3^ in the GC-ML-DG region following WBRT [22]. This effect correlates with deterioration of cognitive functions such as memory and learning [22,23]. Some studies [24] quantified dose–response relationships for white matter and subcortical structures. In white matter, diffusion tensor imaging revealed significant dose- and time-dependent changes (increased mean, axial, and radial diffusivity and decreased fractional anisotropy) that were detectable even at doses of <10 Gy of fractionated WBRT [24]. For subcortical gray matter, WBRT was associated with volume loss in all analyzed structures except the caudate nucleus. This loss was strongly dose-dependent, ranging from 0.16 to 1.37%/Gy (equivalent to 4.9–41.2% losses at 30 Gy), while hippocampal age increased by a median of 11 years one year after radiotherapy [25]. Importantly, WBRT, with diffuse exposure of hippocampi and white matter, produces more pronounced global atrophy than focal SRS, which largely spares distant structures.

Structural changes related to the presence and treatment of brain tumors can lead to cognitive impairment. FreeSurfer allows for the correlation of morphological alterations (e.g., hippocampal atrophy) with neuropsychological test results, as well as investigations of relationships between tumor location and neurological/cognitive symptoms. One FreeSurfer-based study in 162 patients with glioblastoma and high-grade glioma (vs. 127 healthy controls) showed widespread cortical thinning in the hemisphere contralateral to the tumor, and contralesional cortical thickness measures predicted overall survival—a machine learning model differentiated short- from long-term survivors with 83.3% accuracy [26].

Other software packages used to analyze brain structures in neuroimaging studies include FSL and SPM. FSL (FMRIB Software Library, v6.0.7.18) is a comprehensive suite for MRI and fMRI analyses, supporting volumetric analysis, tissue segmentation, and white matter assessments. Its advantages include cross-platform compatibility. However, its tissue segmentation capability may be less detailed than that achieved with FreeSurfer. SPM (Statistical Parametric Mapping, SPM12) is used for tissue segmentation and statistical analysis of brain images. In a prospective head-to-head comparison, SPM-based methods provided the most consistent cross-sectional volumetrics overall, whereas FreeSurfer was more robust for white matter volume segmentation across scanners, and normalization of intracranial volume improved inter-scanner reproducibility for both [27].

#### 3.1.8. Radiomics in Neuro-Oncology

In recent years, an increasing number of studies have explored radiomics, an advanced image analysis approach that converts MRI or PET scans into high-dimensional quantitative features that, combined with clinical variables (e.g., histopathology), are used to build mathematical/machine learning models [28]. The recent interest in the field of radiomics can be attributed to two major factors: Firstly, there has been a notable rise in the utilization of imaging techniques, leading to the generation of substantial volumes of publicly accessible imaging datasets. Secondly, the recent development of advanced data analysis methodologies has enabled the processing and interpretation of these extensive data repositories, thereby opening new avenues for research in this field [29].

The term radiomics refers to the high-throughput extraction and analysis of large amounts of quantitative features from medical images, such as MRI, CT, and PET, using advanced computational algorithms that often incorporate artificial intelligence techniques. These features, which may be hand-crafted or learned through deep learning, capture detailed information about tissue characteristics, heterogeneity, shape, and intensity patterns that are often not discernible by the human eye. Radiomics is a field that combines image data with statistical or machine learning analysis to identify disease-specific imaging signatures. MRI radiomics represents a promising avenue for the management of glioblastoma, as it can be utilized for differential diagnosis, genotype prediction, prognosis, and treatment response prediction in a noninvasive manner [30,31,32].

Radiomics-based investigations in neuro-oncology have been conducted since around 2015, and many radiomics studies in patients with brain tumors employ PET or hybrid PET/MRI techniques [33,34]. In glioma cohorts, FDG PET radiomics has been evaluated as a noninvasive surrogate for the proliferative marker Ki-67, which currently requires tumor biopsy and immunohistochemistry. To date, reported models achieve approximately 80% accuracy in predicting Ki-67 expression levels, suggesting further potential for advancing this approach [33,35]. Clinically, this could be particularly valuable in patients whose tumor location renders biopsy a high-risk procedure for serious neurological complications.

In the prospective review “Recent Deep Learning-Based Brain Tumor Segmentation Models Using Multi-Modality MRI”, the authors demonstrate that the highest accuracy in brain tumor segmentation is achieved by deep learning models that leverage multi-modality fusion (T1/T1c, T2, FLAIR, and, where available, perfusion data and other parametric maps), as these models integrate complementary anatomical and functional information. The best performance is reported in hybrid architectures combining convolutional neural networks with vision transformers (including nnU-Net variants with attention mechanisms), since they capture both local features and the global context, thereby improving delineation of the enhancing core, necrosis, and edema. Model performance, however, depends on careful data preparation (registration and intensity normalization) as well as standardized evaluation protocols (e.g., Dice and Hausdorff) and transparent reporting of configurations. The aforementioned review also emphasizes the dominant role of BraTS benchmarks in method comparison, which facilitates tracking progress but limits generalizability to populations outside these datasets, especially in post-treatment patients. Common challenges include inter-center acquisition variability, missing modalities, class imbalances, the high cost of annotations, and the computational demands of full 3D volumes. According to authors, future directions involve further optimization of hybrid models, semi-supervised and self-supervised learning strategies, more efficient multi-modality fusion, and greater robustness to domain shifts. From a clinical perspective, multi-center prospective validations, datasets reflecting post-treatment cases, and explicit reporting of uncertainty and usage conditions are required to ensure that AI systems can meaningfully support decision-making (e.g., biopsy/resection, SRS/WBRT, and response monitoring) [36].

Currently available datasets are often relatively small and clinically heterogeneous, which favors overfitting and instability of decision thresholds. Annotation quality is limited by variability in diagnostic and therapeutic protocols, segmentation errors, and ambiguous endpoint definitions (e.g., progression vs. pseudoprogression). The lack of prospective, multi-center cohorts restricts the generalizability of findings. Moreover, differences in scanners (vendor and field strength), sequences, and acquisition parameters strongly affect radiomic feature values and model performance. Many studies rely mainly on internal validation, without independent external validation separated by site and time. Calibration methods (e.g., calibration curves and the Brier score) are rarely reported, hindering the assessment of the reliability of predicted probabilities. Clinical utility analyses (e.g., decision curve analysis) and studies evaluating the impact of models on therapeutic decision-making are also scarce. Subgroup analyses (age, sex, biomarkers, and scanner type) are not performed frequently enough, which increases the risk of hidden biases. Meeting regulatory requirements (EU IVDR and FDA SaMD) demands robust clinical evidence, risk management, and post-market surveillance. Legal responsibility for AI-assisted decisions remains unresolved, while privacy and data transfer restrictions continue to impede the development of large, multi-center datasets.

### 3.2. Treatment Strategies

#### 3.2.1. Concomitant Chemoradiotherapy (“Stupp Protocol”)

Surgical resection is the initial standard of care for newly diagnosed glioblastoma whenever feasible. However, malignant glioma cells infiltrate the brain parenchyma beyond the borders visible on imaging, precluding complete eradication. The goals of surgery are to obtain histopathological and molecular diagnoses and to prolong and improve quality of life through maximal safe resection.

The next therapeutic phase consists of concurrent radiotherapy and temozolomide followed by adjuvant temozolomide. In the pivotal trial, the addition of temozolomide to radiotherapy in newly diagnosed glioblastoma produced a statistically significant overall survival benefit with only modest added toxicity. The 2-year overall survival rate was 26.5% with combined radiotherapy and temozolomide versus 10.4% with radiotherapy alone [37].

During the radiotherapy phase, temozolomide is administered at 75 mg/m^2^ daily. After completion of radiotherapy, adjuvant temozolomide is given for 5 days every 28 days (typically six cycles) at 150–200 mg/m^2^/day.

Promoter methylation of the MGMT gene is an important predictive biomarker of responsiveness to temozolomide. Concomitant chemoradiotherapy yields a greater survival benefit in patients with MGMT (O^6^-methylguanine DNA methyltransferase) promoter methylation, a biomarker with both prognostic and therapeutic implications for glioblastoma management.

In one clinical trial, patients with MGMT promoter methylation who received concurrent chemoradiotherapy achieved a median overall survival of 21.7 months compared with 15.3 months among those treated with radiotherapy alone. In patients without MGMT promoter methylation, the survival difference between treatment groups was smaller and not statistically significant [38].

#### 3.2.2. Anti-Angiogenic Therapy

In the recurrent setting, conventional cytotoxic chemotherapy provides only minimal benefit for patients with glioblastoma. Consequently, anti-angiogenic strategies have been explored, notably bevacizumab—a monoclonal antibody directed against vascular endothelial growth factor (VEGF) [39]. One clinical study demonstrated improved progression-free survival and acceptable tolerability with bevacizumab. Efficacy was evaluated for bevacizumab monotherapy and for bevacizumab combined with irinotecan in recurrent glioblastoma. The 6-month progression-free survival (PFS) rates were 42.6% versus 50.3%. Objective response rates were 28.2% versus 37.8%, and median overall survival (OS) was 9.2 versus 8.7 months, respectively [40].

#### 3.2.3. Immune Checkpoint Inhibition

Immune checkpoint inhibitors have produced substantial clinical benefits in multiple malignancies. Unfortunately, for glioblastoma there is still no convincing evidence of meaningful efficacy. In one clinical trial of recurrent glioblastoma (CheckMate 143), patients received nivolumab or bevacizumab. After a median follow-up of 9.5 months, median overall survival (OS) was similar: 9.8 months with nivolumab versus 10.0 months with bevacizumab (hazard ratio [HR] 1.04; *p* = 0.76). The safety profile of nivolumab was similar to that of the anti-PD 1 antibody used in other cancers [41].

Temozolomide added to radiotherapy improves overall survival in glioblastoma, especially in tumors with MGMT promoter methylation. The phase III CheckMate 498 trial evaluated nivolumab plus radiotherapy versus standard concurrent radiotherapy with temozolomide in newly diagnosed MGMT promoter–unmethylated GBM. The primary endpoint was OS. Median OS was 13.4 months in the nivolumab + radiotherapy arm and 14.9 months in the temozolomide + radiotherapy arm (HR 1.31; *p* = 0.0037), demonstrating inferiority of replacing temozolomide with nivolumab in this molecular subgroup [42].

#### 3.2.4. Chimeric Antigen Receptor T-Cell (CAR-T) Therapies

Rapid advances in recent years have led to the regulatory approval of chimeric antigen receptor T-cell (CAR-T) therapies for several hematologic malignancies. This innovative, cell- and gene-based technology harnesses the patient’s immune system—via ex vivo genetically modified autologous T lymphocytes—to recognize and destroy tumor cells.

In glioblastoma, T-cell-based approaches engineered to target tumor-associated antigens represent a promising yet still experimental strategy. CAR-T cells can traffic to the central nervous system and selectively eliminate malignant cells, sparing healthy tissue. Next-generation CAR designs may also deliver immunomodulatory payloads to remodel the immunosuppressive tumor microenvironment. Despite encouraging preclinical data, comparable success in clinical trials has not yet been achieved. Continued investigation of the interplay among the tumor, its microenvironment, and host immune responses to CAR-T therapy is essential to refine this modality and move toward effective immunotherapy for gliomas [42,43].

A phase I clinical trial evaluated intrathecal administration of dual-target CAR-T cells directed against two glioblastoma-associated antigens: epidermal growth factor receptor (EGFR) and interleukin 13 receptor α2 (IL13Rα2). Six patients with recurrent glioblastoma were enrolled. The primary endpoints were safety and determination of the maximum tolerated dose. Reductions in tumor mass on MRI were observed for all participants, although none met the formal criteria for an objective response (ORR). In one patient, regrowth occurred after one month; in another, there was no tumor progression for seven months. One participant discontinued the study early, and in the remaining three patients, tumor size did not return to baseline during the six-month observation period. Notably, tumor shrinkage occurred within 1–2 days after infusion. The study demonstrated preliminary safety and bioactivity of CART EGFR IL13Rα2 cells in recurrent glioblastoma, but evidence of efficacy remains inconclusive and requires confirmation in larger cohorts with longer follow-up periods [44]. Developing multi-target (multi antigen) CAR-T designs may ultimately yield more durable responses by limiting antigen escape mechanisms.

#### 3.2.5. Targeted Therapies

In GBM, increasing attention is being directed toward targeted therapeutic approaches [45]. Multi-omics profiling has identified potentially actionable targets involving the epidermal growth factor receptor (EGFR), vascular endothelial growth factor (VEGF), BRAF V600E mutations, and neurotrophic tyrosine receptor kinase (NTRK) gene fusions.

Although NTRK1/2/3 fusions are rare (≈1% of gliomas), they may act as oncogenic drivers, particularly in higher-grade tumors. Tropomyosin receptor kinase (TRK) inhibitors—such as larotrectinib, entrectinib, and repotrectinib—have demonstrated efficacy in extracranial malignancies harboring NTRK fusions, and their capacity to penetrate the blood–brain barrier makes them attractive candidates in gliomas. Despite encouraging responses in selected patients, the overall effectiveness of TRK inhibition in glioblastoma remains uncertain, and a survival advantage or durable impact on therapeutic resistance has not been definitively established. Further clinical studies are needed to clarify the true role of NTRK fusions and TRK inhibitor therapy in high-grade gliomas, including glioblastoma [46,47].

#### 3.2.6. MRgFUS

A major limitation in the treatment of glioblastoma (GBM) is the blood–brain barrier (BBB), which restricts the penetration of chemotherapeutic agents into tumor tissue. MR-guided focused ultrasound (MRgFUS) with intravenously administered microbubbles has been shown to transiently and repeatedly disrupt the BBB, enhancing the delivery of systemically administered drugs. Combined with chemotherapy, this approach has demonstrated safety and feasibility in clinical settings [48].

##### Decreased Local BBB Permeability

Multiple studies report that MRgFUS-induced BBB opening increases intratumoral delivery of contrast and chemotherapeutic agents, though quantitative data on drug concentration changes remain limited [49]. A recent review of clinical trials [49,50] indicates growing evidence supporting its efficacy in locally reducing BBB permeability. Reported findings include enhanced visibility of molecular tracers in transmission electron microscopy, decreased expression of tight junction proteins and BBB biomarkers (zonulin-1, occludin, and claudin-5), and contrast-enhanced T1-weighted MRI consistent with BBB disruption and restoration [51,52].

##### Promising Drugs

Preliminary studies have investigated MRgFUS with agents such as temozolomide, doxorubicin, pembrolizumab, nivolumab, bevacizumab, carboplatin, paclitaxel, etoposide, panobinostat, and lomustine [53]. Modern protocols incorporate acoustic emissions-guided feedback to titrate sonication and minimize vascular injury, a refinement particularly relevant for patients receiving temozolomide [54].

##### Safety

While certain adverse events—both transient and permanent—are considered acceptable risks in treating advanced CNS tumors, their true incidence and long-term effects, especially in patients with pre-existing neurological deficits, remain uncertain [49,53].

##### Limitations

Despite encouraging results, the main limitation of this approach remains the lack of large-scale randomized controlled trials. The novelty of MRgFUS is reflected in the fact that most clinical studies are ongoing and results are pending. Further longitudinal research with extended follow-up periods is required to confirm safety and therapeutic efficacy, as well as to refine ultrasound parameters [49,50].

## 4. Discussion

### 4.1. Clinical Integration

Recent advances in neuroimaging have been more readily translated into clinical practice than systemic therapies. Multiparametric MRI techniques, such as perfusion, diffusion, and spectroscopy, are increasingly employed for precise tumor delineation, treatment monitoring, and differentiation of progression from therapy-related changes. When available, amino acid PET or hybrid PET/MRI is recommended for detection of recurrence and to guide biopsy targeting, especially in diagnostically ambiguous cases. Radiomics and AI tools further enhance diagnostic precision and may support prognostic modeling, but they remain largely investigational.

On the therapeutic side, maximal safe resection followed by radiotherapy with temozolomide continues to be the standard of care, with MGMT promoter methylation guiding therapeutic stratification. Immune checkpoint inhibitors have failed to demonstrate benefits in unselected glioblastoma, while CAR-T cell therapy is at an early stage of investigation. MR-guided focused ultrasound (MRgFUS) offers a potential strategy to enhance blood–brain barrier permeability and drug delivery, but robust clinical data are lacking. Overall, diagnostic innovations have achieved greater integration into practice than systemic treatments, reflecting the translational barriers in glioblastoma care.

### 4.2. Research Gaps and Future Directions

Several challenges remain. Standardization of advanced imaging protocols and validation of radiomic and AI-based models are required before these tools can reliably inform therapy. Drug delivery across the blood–brain barrier and the immunosuppressive tumor microenvironment continue to limit systemic efficacy; novel carriers, transient barrier-opening techniques, and rational combinations may provide solutions. Targeted therapies for rare actionable alterations (e.g., BRAF mutations and NTRK fusions) need further evidence of clinical benefit in glioblastoma. Immunotherapy approaches require refinement to overcome tumor heterogeneity and antigen escape. Importantly, patient-centered outcomes such as cognitive function and quality of life are underrepresented in trials and should be prioritized. Closing these gaps will depend on multicenter collaboration, harmonized methodologies, and the integration of molecular, imaging, and clinical data into precision-guided care.

## 5. Conclusions

Advanced neuroimaging, particularly multiparametric MRI, novel PET tracers, and AI-driven analysis, has significantly improved diagnostic precision in glioblastoma. However, systemic therapies beyond chemoradiotherapy with temozolomide still provide only limited survival benefit and have not changed population-level prognosis in GBM: Bevacizumab provides mainly symptomatic benefits. Nivolumab requires better stratification and combinatorial strategies. Anti-TRK therapies remain niche, and MRgFUS is still an early-stage, promising technique for enhancing drug delivery. Further progress will depend on prospective, biomarker-driven multicenter trials with clearly defined endpoints (overall survival, quality of life, and benefit calibration) and on rational combination strategies (e.g., immunotherapy plus BBB/angiogenesis modulation) tested within controlled, adaptive research platforms.

## Abbreviations

The following abbreviations are used in this manuscript:

ADC	Apparent diffusion coefficient
AI	Artificial intelligence
BBB	Blood–brain barrier
CAR-T	Chimeric antigen receptor T-cell
Cho	Choline
CNS	Central nervous system
CSF	Cerebrospinal fluid
CT	Computed tomography
DWI	Diffusion-weighted imaging
EGFR	Epidermal growth factor receptor
GBCA	Gadolinium-based contrast agent
GBM	Glioblastoma
^18^F-FDG	^18^F-fluorodeoxyglucose
FLAIR	Fluid-attenuated inversion recovery
IL13R α2	Interleukin 13 receptor α2
MGMT	O^6^-methylguanine DNA methyltransferase
MRgFUS	MR-guided focused ultrasound
MRI	Magnetic resonance imaging
MRS	Magnetic resonance spectroscopy
NAA	N-acetylaspartate
NSF	Nephrogenic systemic fibrosis
NTRK	Neurotrophic tyrosine receptor kinase
PET	Positron emission tomography
PWI	Perfusion-weighted imaging
SRS	Stereotactic radiotherapy
TRK	Tropomyosin receptor kinase
WBRT	Whole-brain radiotherapy
VEGF	Vascular endothelial growth factor
